# Donor Rejection Before Living Donor Liver Transplantation: Causes and Cost Effective Analysis in an Egyptian Transplant Center.

**DOI:** 10.5812/hepatmon.13703

**Published:** 2014-01-02

**Authors:** Mahmoud El-Meteini, Hany Dabbous, Mohammad Sakr, Amany Ibrahim, Iman Fawzy, Mohamed Bahaa, Amr Abdelaal, Mohamed Fathy, Hany Said, Mohamed Rady, Ahmed El-Dorry

**Affiliations:** 1 Ain Shams Center for Organ Transplant (ASCOT), Cairo, Egypt

**Keywords:** Liver Transplantation, Tissue Donors, Tissue Donors

## Abstract

**Background:**

In the living donor liver transplant setting, the preoperative assessment of potential donors is important to ensure the donor safety.

**Objectives:**

The aim of this study was to identify causes and costs of living liver-donors rejection in the donation process.

**Materials and Methods:**

From June 2010 to June 2012, all potential living liver donors for 66 liver transplant candidates were screened at the Ain Shams Center for Organ Transplantation. Potential donors were evaluated in 3 phases, and their data were reviewed to determine the causes and at which phase the donors were rejected.

**Results:**

One hundred and ninety two potential living liver donors, including 157 (81.7%) males, were screened for 66 potential recipients. Of these, 126 (65.6%) were disqualified for the donation. The causes of rejection were classified as surgical (9.5 %) or medical (90.5 %). Five donors (3.9 %) were rejected due to multiple causes. Factor V Leiden mutation was detected in 29 (23 %) rejected donors (P = 0.001), 25 (19.8 %) donors had positive results for hepatitis serology (P = 0.005), and 16 (12.7 %) tested positive for drug abuse. Portal vein trifurcation (n = 9, 7.1%) and small size liver graft estimated by CT volumetric analysis (n = 6, 4.8 %) were the main surgical causes which precluded the donation.

**Conclusions:**

Among potential Egyptian living liver donors, Factor V Leiden mutation was a significant cause for live donor rejection. A stepwise approach to donor assessment was found to be cost-effective.

## 1. Background

Liver transplantation is the only curative treatment for end-stage liver disease. The scarcity of deceased donors in some countries and the absence of cadaveric donors in other countries combined with the urgency of transplantation have resulted in a gradual increase in the number of living donor liver transplantations (LDLTs) (1). In Egypt, the absence of a deceased donor program, till now, made LDLT the only available alternative to end-stage liver disease (ESLD) patients. However, the inherent risks associated with major liver resection in healthy donors add to the importance of preoperative assessment of potential living liver donors for determining optimal graft quality and to ensure the donor safety (2). The Donor safety is paramount, and must not be compromised for the benefit of a given recipient with no exceptions. Thus, a separate donor advocate team should evaluate each potential living donor fitness for donation from all aspects including medical, surgical, and psychological issues (3).

## 2. Objectives

In this study, we attempted to identify and analyze the causes of disqualification of potential Egyptian living liver donors from the process of donation.

## 3. Materials and Methods

### 3.1. Basic Donor Criteria

In General, potential living liver donors accepted for initial evaluation must be ABO compatible with their recipients, age between 18 and 50 years, body mass index (BMI) of 29, with no chronic illnesses, and no previous upper abdominal surgery. 

### 3.2. Donor/Recipient Relationship

All recipients are asked to nominate a family member carrying the basic (minimal) donor criteria. Failure to find a family member with the needed criteria must be documented by the donor advocate team mentioning the medical reasons for rejecting family members from donation in their report. This report is passed to the local ethical committee that might allow, or not, the recipient to nominate a non-related donor, carrying the same nationality, who may be emotionally related as a friend, colleague or lifelong neighbor. The risky nature of this process including the possibility of intimidation and/ or organ trade imposes the necessity of passing through several crucial steps. First, the local ethical committee and an anonymous psychiatrist separately examine all unrelated donors in the absence of the recipient and his or her family as well as any transplant team member to ensure to eliminate any abuse, coercion, or financial benefit. Second, the ethical committee approval for non-related donors is revised and approved, or not, by a higher central committee within the national supreme committee for organ transplant which is the main body supervising and organizing organ transplant in Egypt. Third, after fulfilling the necessary approvals, the donor advocate team starts to evaluate the unrelated donor stepwise, including a second psychological assessment. 

### 3.3. Study Population

During the period between June 2010 and June 2012, 192 potential living liver donors were assessed for 66 liver transplant recipients at the Ain Shams Center for Organ Transplantation (ASCOT). Potential donors were evaluated in 3 phases according to the ASCOT protocol (Table 1). Each phase takes 2 to 3 days. Abnormal findings at any phase would abort the process without further proceeding. Data of all potential donors were retrospectively reviewed to point out the medical and surgical reasons for rejection of donors, and analyzed according to the phase at which the evaluation process was stopped. The donor advocate team is responsible for full explanation of the detailed reasons of rejection from the donation process. 

### 3.4. Informed Consent

All donors retain the right to refrain from donation at any time till the morning of the transplant operation. A senior surgical team member is responsible to explain the contents of informed consent covering all surgical details, hospitalization period, needed medication, all possible complications, and the minimal but possible mortality, which is less than 0.5%. The donor must sign this consent with the recipient in cases with related donors and with 2 close family members in cases with non-related donors.

### 3.5. Ethics

The study was conducted after the approval of the ethical committee and Institutional Review Board (IRB). The study was performed in accordance with the ethical guidelines of the 1975 Declaration of Helsinki. Informed consent was obtained from all study participants. 

### 3.6. Statistics

IBM SPSS statistics software (V. 20.0, 2011) was used for data analysis. Data were expressed as means ± SD for all quantitative measures. For parametric data analysis, the student’s t-test was used to compare the means of the two independent groups. For categorical data analysis, the chi-square test was used to test the associations between two variables, or to compare two independent groups.

A P value > 0.05 was considered insignificant, a P-value < 0.05 was considered significant, and a P value < 0.01 was considered highly significant. A multiple regression analysis was used to identify independent parameters that could predict dependent variables.

**Table 1.** Phases of Preparation of Living Donor According to the ASCOT Protocol

Phase	Test
**Phase I**	A) Full history and physical examination
	B) Laboratory investigations
	- ABO blood grouping, CBC, CRP
	- Liver profile, Renal profile, Coagulation profile, TSH, and lipid profile.
	- Factor V Leiden mutation.
	- Fasting blood sugar, Iron, and ferritin.
	- Schistosoma Ab Titers, circulating bilharzial antigen, and rectal snip positive antibody titer.
	- HCV Ab/ HBV s Ab and Ag/ HBc Ab/ HIV (If HBV s Ab is –ve, HBV vaccine is given at 0, 1, and 6 months)
	- urine analysis for drug abuse and stool analysis
	C) Imaging studies
	- Abdominal ultrasound with Doppler study.
	- Chest X-ray.
	- Pelvic US for females and sono-mammography if >30 years.
**Phase II**	A) Viral Markers
	HAV IgM/ HAV total/ HBe Ab/ HBe Ag/ HBc IgM/ EBV IgG and IgM/ CMV IgG and IgM/ HCV RNA/ HBV DNA, HSV (IgG – IgM),Varicella-Zoster (IgG – IgM).
	B) Tumor Markers
	- Alfa-feto protein/ Carcinoembryonic antigen/ Cancer antigen 19-9.
	- Cancer antigen 125 in females.
	- Cancer antigen 15-3 (males and females)/ Prostatic specific antigen in males) above 40 yrs.
	C) Cardio-pulmonary Consultations
	D) Protein C and S- Antithrombin III
**Phase III**	A) Imaging studies:
	- Spiral Triphasic Computed Tomography with Venography and portography
	- Computed tomography Volumetry: graft recipient weight ratio "GRWR" and Residual liver volume (RLV).
	- Magnetic Resonance Cholangiopancreatography (MRCP).
	B) Consultations: Psychiatry, Anesthesia,
	C) Liver biopsy

## 4. Results

### 4.1. Donor Demographics

A total of 192 potential living liver donors, 35 females and 157 males, were screened and evaluated at the ASCOT for 66 liver transplant candidates (Table 2). 

**Table 2.** Demographic Data of 192 Potential Living Liver Donors

	Accepted	Rejected	P value	Significance
**Number**	66	126		
**Gender (Male/Female)**	51/15 (77.2/22.8)	106/20 (84.1/15.9)	0.170	None
**Age, Mean ± SD, y**	29.8 ± 6.6	29.9 ± 6.5	0.894	None
**Age, range, y**	19-47	18-48		
**BMI , mean ± SD, kg/m^2^** ^**a**^	24.6 ± 3.2	25.3 ± 5.1	0.287	None
**BMI, range, kg/m** ^**2**^	18.7-29.7	19.4- 31.7		
**Relationship to the recipient**				
Mother	2	3		
Son	8	9		
Daughter	4	3		
Brother	5	18		
Sister	3	2		
Spouse	2	3		
Other relative (cousin and nephew)	2	12		
Unrelated	40	76		

^a^Abbreviation: BMI, body mass index.

### 4.2. Medical and Surgical Causes of Rejection

Reasons for disqualification of 126 donors were classified as either surgical (n = 12) (9.5%) or medical (n = 114) (90.5%) (Table 3). Factor V Leiden mutation was the most common cause of rejection of potential donors, followed by positive results for hepatitis serology, substance abuse, and unsafe portal venous or biliary anatomy. Factor V Leiden mutation, detected in 29 donors (23%), and positive results for hepatitis serology, detected in 25 donors (19.8%), were the main medical contraindications for donation. Hepatitis B core antibody (HBcAb) positive donors were accepted in cases of HBcAb positive recipients, if they were willing to receive antiviral therapy lifelong. 

Portal vein trifurcation type IV (n = 9, 7.1%) was the most common regarding surgical causes. Two donors with abnormal biliary anatomy variants were rejected due to the drainage of the right liver by 3 very small separate right ducts, and to avoid injury to the left duct.

**Table 3.** Reasons for Rejection of Potential Donors

Phase	Reason for Rejection	No. (%)
**Phase I**	Hypercoagulability due to FV Leiden mutation	29 (23)
	Positive results for hepatitis serology	25 (19.8)
	- HCV	10 (7.9)
	- HBcAb	15 (11.9)
	Substance abuse	16 (12.7)
	Abnormal liver enzymes	10 (7.9)
	Dyslipidemia	9 (7.1)
	Positive bilharzial serology	4 (3.2)
	Thyrotoxicity	1 (0.8)
**Phase II**	Decreased protein C value	3 (2.4)
	Decreased antithrombin III value	2 (1.6)
**Phase III**	Small-size liver by volumetry	6 (4.8)
	Unsafe anatomy	12 (9.5)
	- Portal vein variants (trifurcation)	9 (7.1)
	- Biliary anomalies	2 (1.6)
	- Nephrectomy	1 (0.8)
	Abnormal liver histology (by biopsy)	11 (8.7)
	- Steatosis	9 (7.1)
	- Inflammation	1 (0.8)
	- Granuloma	1 (0.8)

Four donors (3.1%) were rejected for multiple causes during phase I due to positive results for hepatitis serology, and drug abuse. 

**Figure 1.**
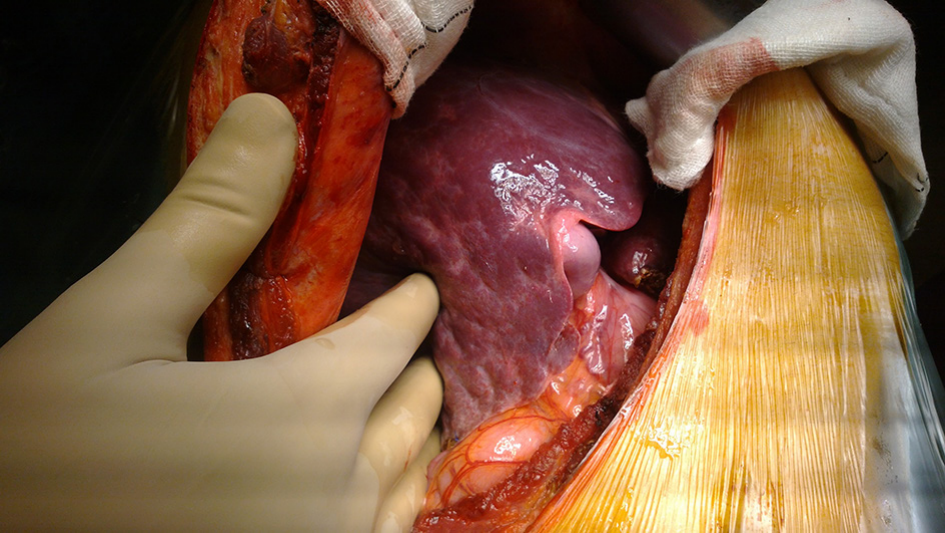
Intraoperative Picture of a Rejected Donor Liver Despite normal pathology by liver biopsy before the operation.

One donor was rejected intraoperatively due to irregular liver borders, and a nodular surface despite a normal liver profile, negative serology results for schistosomiasis, negative results for virological study, absence of drug abuse, and a preoperative biopsy showing minimal fibrosis (Figure 1). The donor was accepted because the biopsy showed only mild fibrosis in 1 of 7 portal tracts with no necro-inflammatory cells. However, the surgeons felt that the liver does not look good intraoperatively, and the intra-operative wedge biopsy proved the presence of mild fibrosis in most of the portal tracts. 

### 4.3. Cost Effectiveness

The cost of each phase of donor preparation was calculated in the US dollars (Table 4). Phase III was more expensive compared to phases II and I. 

**Table 4.** Costs of Donor Evaluation

Phase	Costs (in the US dollars)
**Evaluation phase**	55
**Phase I**	300
**Phase II**	400
**Phase III**	700
**Total**	1455

## 5. Discussion

The most important issue in LDLT is donor safety, and the ability to donate the appropriate graft size for the recipient. The risks of mortality and morbidity with live donor right hepatectomy have been previously estimated as 0.4% and 35%, respectively (4). Only one-third of potential donors are accepted as candidates for the procedure (5). Some centers have used a 4-phase approach to assess live liver donors (6, 7), while another center used a 5-step evaluation protocol. In this study, we tried to determine contraindications to donation early before conducting expensive and invasive tests. Phase 1 represents the highest number of rejected donors before proceeding to the final phase, which involves anatomical and pathological assessment of the graft. This evaluation protocol is close to the protocols used at Cairo University, Kyoto University in Japan (5), and the University of Heidelberg in Germany (8).

In a previous series, 68.2% of potential donors were excluded early, and only 23% of those who had complete work-up underwent the operation. Both donor and recipient reasons for rejection were included (9). However, our study was mainly concerned with reasons for donor rejection. In addition, our study is unique, because it showed that the main reason for rejection was Factor V Leiden mutation. In another study, 33% of potential donors were rejected (10). In a series of patients, 11% of potential donors were not accepted due to fatty liver and abnormal liver enzymes, which is in agreement with our findings (eight with fatty liver, and nine with abnormal transaminases of 126 donors) (11). Renz and Roberts accepted only 13% of potential donors, with 23% rejected for medical reasons, and 20% rejected for psychosocial reasons (12). In our study, 51% of donors were rejected early. The remaining 49% proceeded to the third phase, and 14.6% were excluded at this phase. In another series, of 126 donors, 69% were disqualified due to incompatible blood type, steatosis with low graft-to-recipient weight ratio, and positive results for hepatitis serology (6).

Although the donor rejection percentage (65.6%) in our study was similar, ABO-incompatible potential donors were not enrolled in our study. Nevertheless, Factor V Leiden mutation (23%) was the most significant cause of rejection in our study, which is incomparable to any other series, and represents a new finding requiring broad investigation. Factor V Leiden mutation is the most common etiological factor in Egyptian patients with Budd-Chiari Syndrome (13). Our data also showed a higher percentage of positive results for hepatitis serology compared to the previous studies (7.9% HCV and 11.9% HBcAb), which is explained by a higher prevalence of viral hepatitis in Egypt than other countries. We also had more potential donors rejected for drug abuse (12.7%), fewer donors with steatosis (7.9%), and a similar proportion of donors with small-for-size livers (4.8%) compared to the study mentioned above. Valentin-Gamazo and colleagues found only 14% of potential donors to be suitable, and excluded 67% with positive results for hepatitis markers or blood incompatibility (14). In another study, 56.5% of potential donors were excluded. Positive results for hepatitis serology and ABO incompatibility were the main contraindications to donation (15). Pascher et al. reported that 39.9% of potential donors were declined because of blood group incompatibility or obvious contraindications, 18% had steatosis of more than 10%, and 7.9% showed psychological contraindications (16). In our center, we accepted HBcAb-positive donors provided for a recipient who was also HBcAb-positive. In a 2005 study by Nadalin et al. 108 of 730 donors (15%) were considered suitable. Liver biopsy revealed a positive finding in 31 of 144 candidates, including 21 cases of steatosis, and 10 cases of non-steatotic hepatopathy (17). In our study, 94 potential donors proceeded to phase 3 and underwent liver biopsies. Eleven (8.7 %) subjects had positive pathological findings and were rejected, 9 (7.1 %) due to steatosis of more than 15%, and 2 due to non-steatotic hepatopathy (non-specific inflammation in one case, and non-specific granuloma in the other). Though we routinely take biopsy of all accepted donors, one donor was rejected intraoperatively because of an irregular liver border and nodular surface. Fifty cases of LDLT were performed at the West China Hospital, Sichuan University. The evaluation process yielded 10 cases with a volume of remnant liver of more than 30%, which would make a potential donor ineligible (15). In our study, 4.8 % of cases were excluded due to small-size liver.

Schroeder et al. reported that 40.8% of potential donors were rejected due to unfavorable anatomy, mostly inappropriate hepatic volumes. Two candidates were rejected due to the presence of 3 or more vascular and biliary variants (18). However, the proportion of potential donors rejected due to unfavorable anatomy in our study was only 9.5%. This was due to portal vein variants (trifurcation) in 9 (7.1%) cases, biliary anomaly in 2 (1.6 %) cases, previous major operation (nephrectomy) in one (0.8 %) case, and small-size liver in 6 (4.8 %) cases.

We believe that the stepwise approach to donor preparation used in our study is cost effective. In a previous study, the reported costs of donor evaluation were 567 € (Euro) for step 1, 794 € for step 2, 1462 € for step 3, 185 € for step 4, and 1581 € for step 5, for a total cost of 4589 € (14). In our study, the evaluation procedure was less expensive. The cost of the complete evaluation procedure was $1455 (The US dollars, approximately 1110 €), including $55 for the preliminary evaluation phase, $300 for step 1, $400 for step 2, and $700 for step 3. Professional fees were not included, similar to the previous study. Though, this cost may appear low, in a country with limited resources and a university-based transplant program serving low-income patients lacking health insurance coverage, this cost is considered high, especially if more than one donor is tested for a single recipient. In conclusion, Factor V Leiden mutation is a new significant cause of rejection among Egyptian potential living liver donors. A stepwise approach to donor evaluation is cost-effective. Implementation of cadaveric liver transplantation would spare the expense of donor preparation as well as the surgery risk imposed on donor. Because of the very low rate of donor rejection as well as abnormal findings in phase 2, and a much higher number of rejections after CT scanning, we would change our protocol, so that CT scanning can be performed much earlier to check for potential anatomical and size contraindications for liver donation in potential donors.
